# MiR-200b is upregulated in plasma-derived exosomes and functions as an oncogene by promoting macrophage M2 polarization in ovarian cancer

**DOI:** 10.1186/s13048-021-00826-9

**Published:** 2021-06-02

**Authors:** Jun Xiong, Xiaoju He, Yuanyuan Xu, Wei Zhang, Fen Fu

**Affiliations:** Department of Obstetrics and Gynecology, The Second Affiliated Hospital of Nanchang University, No.1 Minde Road, Donghu District, 330006 Nanchang, Jiangxi China

**Keywords:** Ovarian cancer, MiR-200b, Macrophage polarization, Kruppel like factor 6

## Abstract

**Background:**

Ovarian cancer is the seventh most common cancer in women and the second most reason of gynecologic cancer-related death. Growing evidence showed that exosomal miRNA plays a crucial role in the progression of ovarian cancer.

**Methods:**

Exosomes were identified using nanoparticle tracking analysis, transmission electron microscopy and marker proteins detection. The levels of mRNA and proteins were ensured by qRT-PCR and western blot, respectively. Immunofluorescence, flow cytometry and ELISA assay were carried out to analyze macrophages polarization. CCK-8 and Transwell assay were used to measure the cell viability and invasion of ovarian cancer cells. The interaction of miR-200b and Kruppel like factor 6 (KLF6) was ensured by using luciferase reporter assay.

**Results:**

Here, we obtained plasma-derived exosomes successfully, and proved that miR-200b was increased in the exosomes of ovarian cancer patients. Subsequently, our data showed that increasing of miR-200b could promote macrophage M2 polarization, but inhibit M1 polarization. miR-200b-overexpressed macrophages-conditioned medium notably enhanced the cell viability and invasion of ovarian cancer cells. Moreover, increasing of miR-200b inhibited KLF6 expression, while decreasing of miR-200b promoted KLF6 expression. Overexpression of KLF6 recused miR-200b-induced macrophage polarization toward M2, and the inhibitory effect of miR-200b on M1 polarization.

**Conclusions:**

Overall, our results demonstrated that miR-200b was highly expressed in the plasma-derived exosome of ovarian cancer patients, and promoted the proliferation and invasion of ovarian cancer cells through inducing macrophage M2 polarization by suppressing KLF6 expression. Our results suggested that miR-200b might be a novel target for ovarian cancer treatment.

## Background

Ovarian cancer is the seventh most common cancer in women, and it is the second most reason of gynecologic cancer-related death and the eighth most common reason of cancer-related death in women globally. The five-year survival rate of ovarian cancer patients is approximately 47 % [1, 2]. Epithelial ovarian cancer (EOC) is the major type of ovarian cancer, accounting for 85-90 % of total case. According to the origin, pathogenesis, risk factors, prognosis and molecular alterations of ovarian cancer, it can be divided into five subtypes [3, 4]. Generally, early in ovarian cancer patients is not easy to be found, hence, the majority of patients with clinical advanced stage when they were diagnosed. For the patients with advanced ovarian cancer, surgical treatment and chemotherapy are the major therapeutic methods, while the resistance to chemotherapeutic drugs greatly declines the outcome of the patients [5, 6]. Thereby, it is very necessary to find a new target for ovarian cancer treatment.

Exosomes are one of the type of vesicles with 30–150 nm of diameter, which can be released by almost all cell types such as tumor cancer cell and macrophage. Exosome are an important messenger among different cells [7]. In recent years, more and more studies have indicated that exosomes play a crucial role in the development of multiple diseases, including ovarian cancer [8]. It was reported that exosomes could transmit messages to target cells through carrying microRNA (miRNA), protein and other moleculars, thus to involve in many biological process like communication in intercellular, cell differentiation and immune modulation [9]. Growing evidence that miRNA, a class of endogenous non-coding RNA, can be acted as one of the index in tumor detection and a potential target for tumor treatment [10]. For instance, Zhang et al. demonstrated that miR-337-3p is decreased in EOC tissues. MiR-337-3p promoted EOC cell cycle arrest in G0/G1 phase and apoptosis and inhibited cell proliferation, suggesting miR-337-3p is a potential therapeutic target of EOC [11]. Recently, Pan et al. found that numerous miRNAs are abnormally expressed in the plasma-derived exosome of the EOC patients, such as miR-200b [12]. However, whether exosomal miR-200b participates in regulating the development of ovarian cancer remains unclear.

Some studies indicated that exosomal miRNAs could affect the progression of cancer through mediating the interaction of cancer cells and immune cells [13]. Macrophages are an important class of immune cells in tumor microenvironment. In accordance with different extracellular environment, macrophages can differentiate into M macrophages and M2 macrophages [14]. It was proved that M1 macrophages suppress cancer progression through secreting pro-inflammatory cytokines, whereas M2 macrophages promote cancer progression via secreting anti-inflammatory cytokines [15, 16]. A recent study indicated that miR-21 is increased in ovarian cancer tissues, and it enhances the chemoresistance of ovarian cancer cells through facilitating macrophage M2 polarization, indicating the crucial role of miRNA in the interaction of macrophage and ovarian cancer cells [17]. In this present study, we also proved that miR-200b was highly expressed in the exosomes from the plasma of EOC patients, and indicated that miR-200b promoted ovarian cancer cell proliferation and invasion via promoting macrophages M2 polarization and inhibiting M1 polarization by repressing Kruppel like factor 6 (KLF6) expression. Our data revealed firstly that the regulatory mechanism of miR-200b in ovarian cancer proliferation, and suggested that miR-200b might a potential target for ovarian cancer treatment.

## Materials and methods

### Exosome isolation and identification

All participants provided the written informed consent. This study was approved by the Second Affiliated Hospital of Nanchang University. For exosome isolation, peripheral blood samples were taken from three healthy women, and six EOC patients who accepted with therapy in our hospital. Then, ExoQuick™ Exosome Precipitation Solution (System Biosciences, Mountain View, Calif) was utilized to extract exosomes from the plasma in accordance with the manufacture’s introduction. Transmission electron microscopy (TEM) was utilized to analyze the morphology of exosomes, and the size and concentration of exosomes was ensured by nanoparticle tracking analysis (NTA) on a NanoSight NS300 (Malvern Instrument Ltd.). Besides, the expression of the markers of exosomes, including lysosome-associated membrane proteins 1 (LAMP-1) and tumour susceptibility gene 101 (TSG101), were measured by using western blot.

### Cell culture and treatment

Human monocytic cell line THP-1 was purchased from the American Type Culture Collection (ATCC, USA). THP-1 cells were cultured in RPMI-1640 medium containing 10 % fetal bovine serum (FBS, Gibco, New York, USA), 1 % streptomycin and penicillin (Gibco), and 0.05 nM β-mercaptoethanol (Gibco). 50 ng/ml of phorbol-12-myristate 13-acetate (PMA, Sigma-Aldrich, Missouri, USA) was used to induce the differentiation of THP-1 cells toward macrophages. After incubation with PMA for 48 h, the THP-1 cells were collected, named as M0 macrophage, and then were used in subsequent studies. In our present study. To explore the effect of miR-200b on macrophage polarization, we obtained miR-200b mimic and mimic negative control (mimic NC), and miR-200b inhibitor and inhibitor negative control (inhibitor NC) from RiboBio (Guangzhou, China). Besides, we obtained the lentiviruses packaged with KLF6 overexpression vector (LV-KLF6) and the lentiviruses packaged with empty vector (LV-NC) from BrainVTA (Wuhan, China). Here, 50 nM of miR-200b mimic, 50 nM of mimic NC, 100 nM of miR-200b inhibitor and 100 nM of inhibitor NC were transfected into M0 macrophages using the transfection reagent Lipofectamine 2000 (Invitrogen, Carlsbad, USA) according to the protocol. After 24 h of cell transfection, qRT-PCR, western blot and other experiments were carried out.

Human ovarian carcinoma cell line OVCAR-3 also was obtained from ATCC, and the cells were maintained in RPMI-1640 medium supplemented with 10 % FBS and 1 % streptomycin and penicillin. After 24 h of miR-200b mimic and mimic NC transfection, the conditioned cell culture medium of M0 macrophages, mimic NC-transfected M0 macrophages and miR-200b mimic-transfected M0 macrophages were collected, and then were used to culture OVCAR-3 cells. PBS-treated OVCAR-3 cells acted as control group. At 24 h later of incubation, cell proliferation and cell invasion were measured. In our study, all cells were cultured at 37 °C in an incubator with 5 % CO_2_.

### Western blot

The expression of LAMP-1 and TSG101 in exosomes, and the expression of KLF6 in M0 macrophages were measured by using western blot. RIPA lysis buffer was used to isolate total protein. Equal quality 25 µg of cellular protein from each group were separated on the 12 % SDS-PAGE gel, and then were transferred to a PVDF membrane. After that, membranes were blocked with 5 % non-fat mile for 1 h at room temperature and subsequent primary antibodies overnight at 4 °C. The primary antibodies were as follows: anti-LAMP-1 antibody (1:1000, Cell Signaling Technology, Boston, MA, USA), anti-TSG101 antibody (1:1000, Cell Signaling Technology) and anti-KLF6 antibody (1:1000, Cell Signaling Technology). After incubation with primary antibodies, the membranes were maintained with secondary antibodies (1:3000, Abcam) for 1 h at room temperature. At last, the membranes were maintained with the enhanced chemiluminescence reagent (Merck Millipore, Missouri, USA) to detect the protein bands. The relative expression level of KLF6 was analyzed utilizing Image J software (National Institutes of Health, Maryland, USA). Here, β-actin served as the internal reference of KLF6.

### QRT-PCR

Total RNA was extracted from M0 macrophages using TRIzol reagent (Invitrogen) in accordance with the manufacture’s introduction. Then, the concentration of total RNA was ensured utilizing the NanoDrop 2000, and 1 µg of total RNA was added into the reaction system of reverse transcription. PrimeScript RT reagent kit (TaKaRa) was used to synthesis complementary DNA according to the manufacture’s protocol. After that, real-time PCR was implemented on ABI 7300HT instrument (Applied Biosystems) using the SYBRR Green Supermix kit (TaKaRa) in accordance with the protocol. The relative expression levels of KLF6, iNOS and Arg-1 mRNAs were normalized to *GAPDH*, and U6 served as the internal reference of miR-200b.

### Immunofluorescence analysis

M0 macrophages were planted at the density of 1 × 10^5^ cells per well into a 24-well plate and subsequent transfected with miR-200b mimic and mimic NC for 24 h. Next, the cells were fixed with 4 % paraformaldehyde (Solarbio, Beijing, China) for 30 min at 4 °C and subsequent incubated with 0.5 % Triton X-100 for 20 min at room temperature. After that, the cells were incubated with 2 % BSA for 30 min followed by primary antibodies, including anti-CD86 antibody (1:200, Abcam, Cambridge, MA, USA) and anti-CD206 antibody (1:200, Abcam) overnight at 4 °C. Next day, the cells were maintained with secondary antibodies, including Alexa Fluor-488-conjugated goat anti-rabbit IgG antibody (1:1000, Abcam) and Alexa Fluor-674-conjuated goat anti-mouse IgG antibody (1:1000, Abcam), for 1 h at room temperature in the dark. Next, the cells were stained with DAPI reagent for 5 min in the dark. At last, the CD86-positive and CD206-positive cells were observed under a confocal microscope (Leica). The red and blue images were overlaid to create the merge images.

### Flow cytometry

M0 macrophages were planted at the density of 1 × 10^5^ cells per well into a 24-well plates. After 24 h of cell transfection and lentivirus infection, the cells were collected for flow cytometry. The cells were maintained with PE-labeled CD206 and FITC-labeled CD86 antibodies for 30 min at room temperature. Next, the stained cells were analyzed using a BD Accuri™ C6 cytometer (BD Biosciences).

### ELISA assay

ELISA assay was carried out to measure the concentration of interleukin-1β (IL-1β) and C-C motif chemokine ligand 17 (CCL17) in cell culture supernatants. ELISA assay was accomplished according to manufacturer’s protocols of human IL-1β ELISA kit (R&D systems, Minneapolis, USA) and human CCL17 ELISA kit (R&D systems).

### CCK-8 assay

Cancer cells were planted at the density of 6 × 10^3^ cells per well into 96-well plates. M0 macrophages-, mimic NC-transfected M0 macrophages- and miR-200b mimic-transfected M0 macrophages-conditioned medium were used to culture cancer cells. At 24 h later of cell culture, the cells were maintained with 10 µl of CCK solution (Solarbio) for another 2 h at 37 °C. Then, the optical density of each well at 450 nm were measured using a microplate reader (Elx800; BioTek Inc., North Brunswick, NJ, USA).

### Transwell invasion assay

The bottom of the upper chambers of 24-well transwell chambers (Corning Incorporated, Corning, NY, USA) were pre-coated with matrigel. 1 × 10^4^ cancer cells were seeded into the upper chamber, and were cultured with serum-free RPMI-1640 medium. The 1:1 mixture of macrophages-conditioned medium and fresh RPMI-1640 medium containing 15 % FBS was added into the lower chambers. At 24 h later of cell culture, the invaded cancer cells were fixed with 10 % methanol and then stained with 0.1 % crystal violet. At last, the stained cancer cells were observed using a microscope.

### Luciferase reporter assay

The binding sites between miR-200b and KLF6 3’-UTR were predicted using starBase v2.0 database. The wild-type (wt) and mutant (mut) gene sequences of KLF6 3’-UTR containing the binding sites with miR-200b were sub-cloned into p-GL3 vector. Then, KLF6 3’-UTR wt vector or KLF6 3’-UTR mut vector were co-transfected into HEK293T cells with miR-200b mimic or mimic NC. At 24 h later of transfection, the luciferase activity of cells were measured. In the luciferase reporter assay, we used two negative control (mimic NC and luciferase reporter lacking the KLF6 3'-UTR).

### Statistical analysis

All data analysis were performed using SPSS 22.0 software, and expressed as mean ± SD. The significant difference between two independent groups was analyzed using Student *t-*test, and the significant difference among multiple groups was analyzed using one-way ANOVA. The value of P lower than 0.05 as considered statistically significant.

## Results

### miR-200b was highly expressed in the plasma-derived exosome of EOC patients

**Fig. 1:**
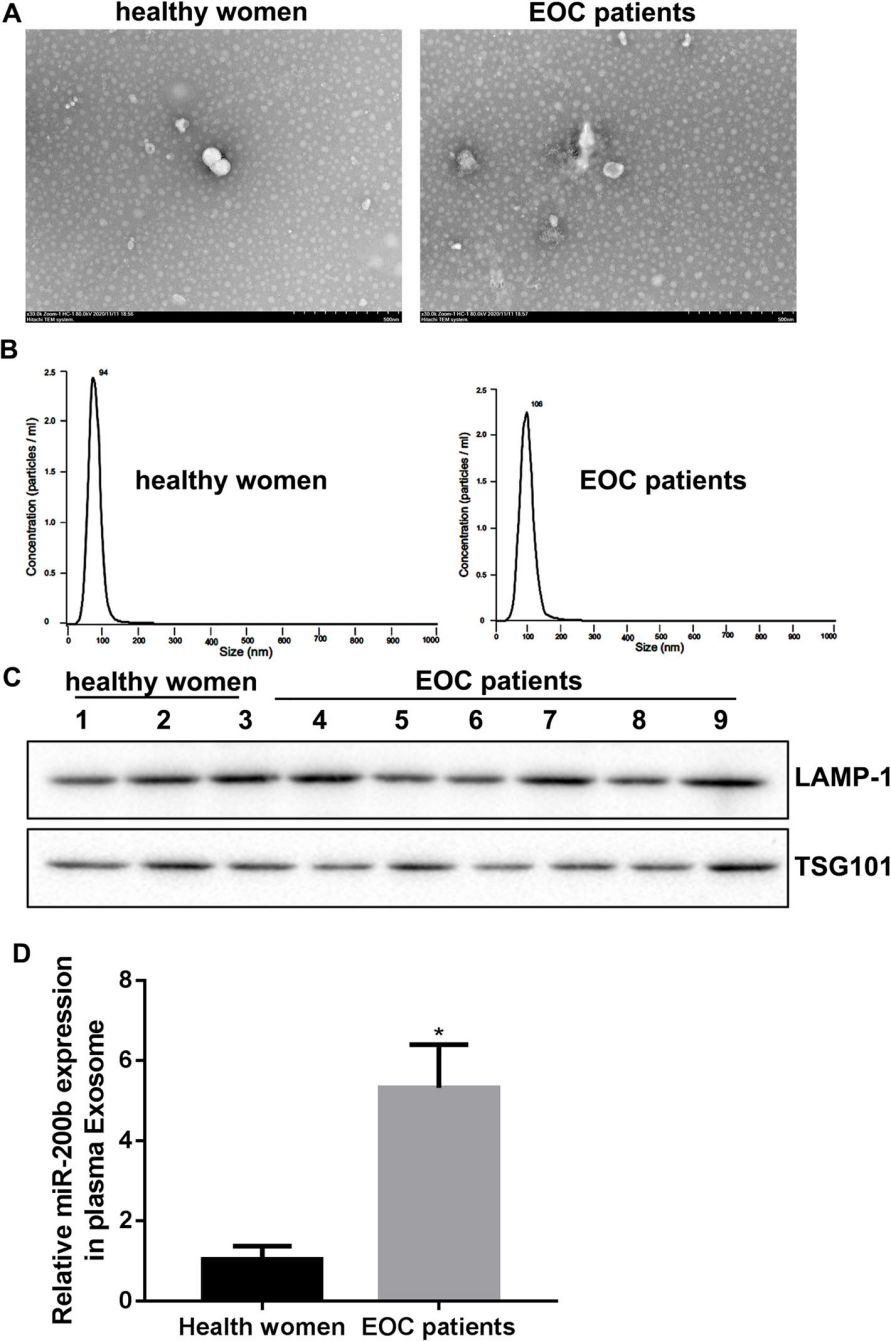
The expression of miR-200b in the plasma-derived exosomes. Exosomes were isolated from the plasma of healthy women and EOC patients. **a** TEM was used to observe the morphology of exosomes. **b** NTA was used to analyze the size and concentration of exosomes. **c** The markers of exosomes, including LAMP-1 and TSG101, were detected by western blot. **d** qRT-PCR was carried out to measure the level of miR-200b in exosomes. *P < 0.05 compared with Health women

To verify the expression levels of miR-200b in the plasma-derived exosomes of healthy women and EOC patients, we isolated the exosomes from the plasma of 3 cases of healthy women and six cases of EOC patients. The typical circle and saucer-like structures characteristic of exosomes were observed using a TEM (Fig. 1a), and the diameters of exosomes lower than 100 nm was identified by NTA (Fig. 1b). In addition, we found that the markers of exosomes, including LAMP-1 and TSG101, expressed in the plasma-derived vesicle (Fig. 1c). Importantly, our results showed that the level of miR-200b was higher in the plasma-derived exosomes of EOC patients than that in the plasma-derived exosomes of healthy women (Fig. 1d).

### miR-200b promoted macrophage M2 polarization, while inhibited M1 polarization

**Fig. 2:**
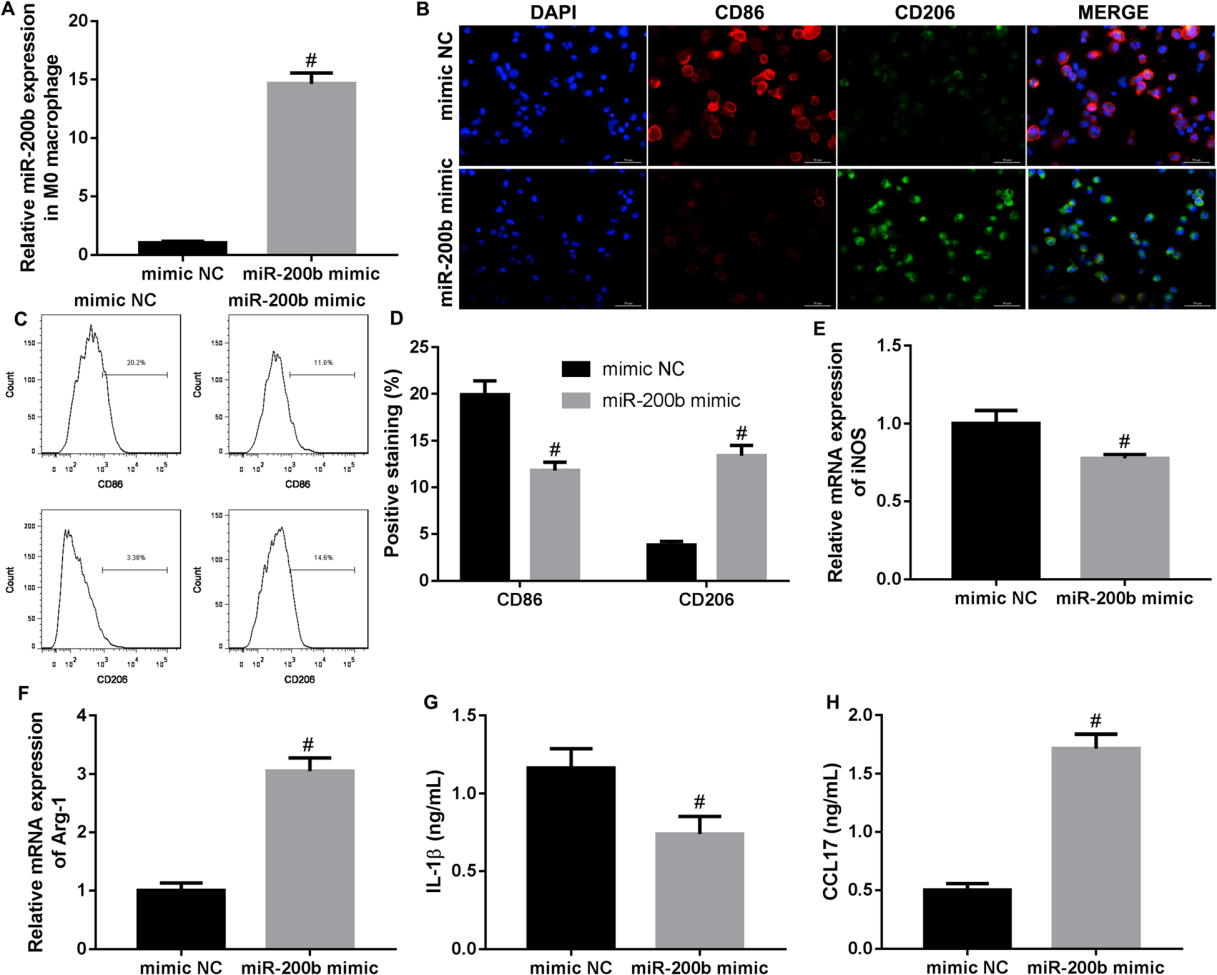
The effect of miR-200b on macrophages polarization. miR-200b mimic and mimic NC were transfected into M0 macrophages. At 24 hours after transfection, **a** qRT-PCR was performed to examine the expression of miR-200b in the cells. **b** and **c** Immunofluorescence and flow cytometry were utilized to detect the number of CD86-positive cells and CD206-positive cells. **d** The percentages of CD86-positive and CD206-positive macrophages were analyzed in accordance with the results of flow cytometry. **e** and **f** The levels of iNOS mRNA and Arg-1 mRNA were measured by qRT-PCR. **g** and **h** The concentrations of IL-1β and CCL17 in cell supernatant of macrophages were measured by ELISA assay. ^#^*P* < 0.05 contrasted with mimic NC

To explore the effect of miR-200b on the polarization of macrophage, we designed the miR-200b mimic and mimic NC. Our data showed that the expression of miR-200b was significantly promoted by miR-200b mimic treatment in M0 macrophages (Fig. 2a). Subsequently, we detected the polarization of macrophages toward M2 and M1. The results of immunofluorescence indicated that the number of CD86-positive cells (M1-polarized macrophages) was notably reduced, whereas the number of CD206-positive cells (M2-polarized macrophages) was increased in M0 macrophages by miR-200b mimic treatment (Fig. 2b). Flow cytometry results also demonstrated that the percentage of CD86-positive cells was downregulated, but the percentage of CD206-positive cells was significantly upregulated by miR-200b mimic treatment (Fig. 2c and d). Moreover, we further found that the level of iNOS mRNA was lower, while the level of Arg-1 mRNA was higher in the M0 macrophages transfected with miR-200b mimic compared to the M0 macrophages transfected with mimic NC (Fig. 2e, f). The concentration of M1 macrophages-related cytokine (IL-1β) was declined, and the concentration of M2 macrophages-related cytokine (CCL17) was increased in the cell supernatants of miR-200b mimic-treated M0 macrophages (Fig. 2g, h). In summary, miR-200b promoted macrophage polarization toward M2, and inhibited macrophage polarization toward M1.

### miR-200b facilitated EOC cells proliferation and invasion

**Fig. 3:**
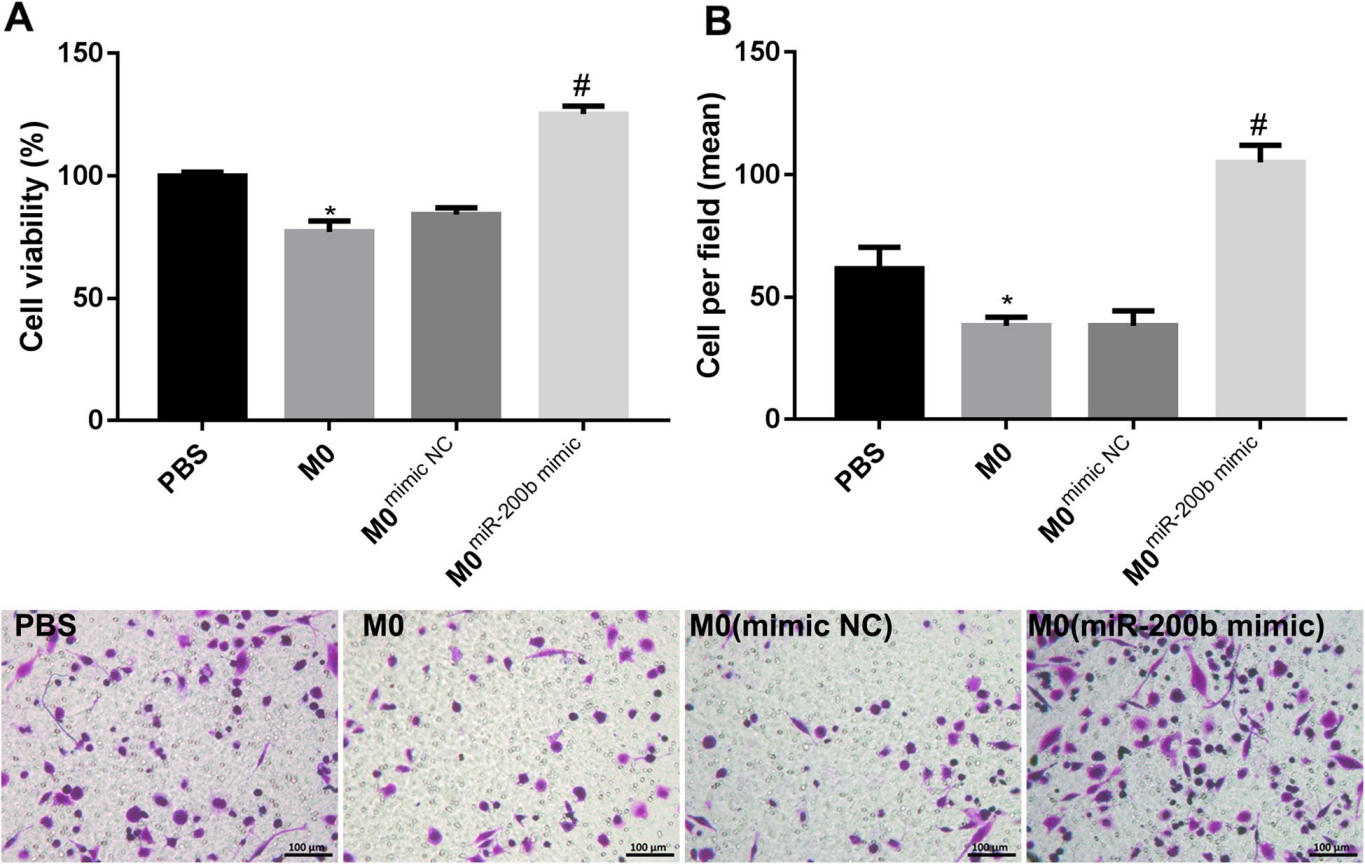
miR-200b regulated ovarian cancer cell proliferation and invasion through affecting macrophage polarization. M0 macrophages were transfected with nothing, mimic NC or miR-200b mimic. Then, the culture medium of macrophages were collected and then were used to culture ovarian cancer cells. At 24 h later of incubation, **a** CCK-8 assay was carried out to detect the cell viability of the cancer cells. **b** Transwell invasion assay was utilized to examine the invasion of cancer cells. *P < 0.05 compared with PBS. ^#^*P* < 0.05 contrasted with M0^mimic NC^

Furthermore, at 24 h after miR-200b mimic and mimic NC transfection, we collected the culture medium of M0 macrophages, mimic NC-treated M0 macrophages and miR-200b-transfected M0 macrophages. EOC cells were incubated with above cell culture medium for 24 h. CCK-8 assay demonstrated that M0 macrophages-conditioned medium suppressed the cell viability of EOC cells, while miR-200b increasing notably facilitated the cell viability of EOC cells (Fig. 3a). In addition, M0 macrophages-conditioned medium inhibited the invasion of EOC cells, but miR-200b-overexpressed M0 macrophages-conditioned medium markedly exacerbated the invasion of EOC cells (Fig. 3b). Overall, miR-200b promoted EOC cells proliferation and invasion through inducing macrophage M2 polarization.

### miR-200b regulated macrophage polarization through inhibiting KLF6

**Fig. 4:**
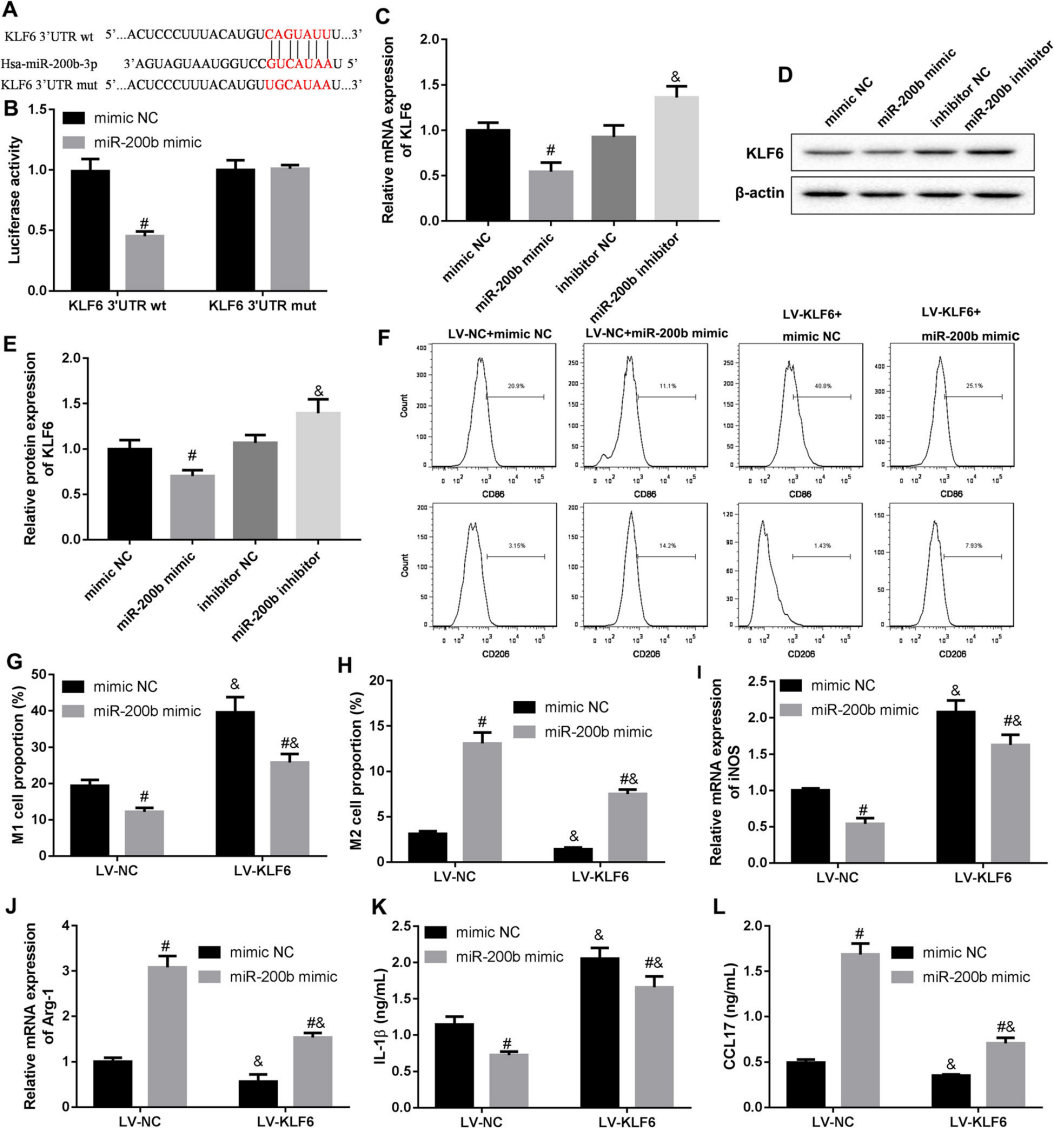
miR-200b regulated macrophage polarization via targeting KLF6. **a** and **b** The binding sites between miR-200b and KLF6 3’-UTR was predicted according to starBase v2.0 database, and the interaction of the two molecules was ensured by luciferase reporter assay. **c** and **e** M0 macrophage was transfected with miR-200b mimics and infected with the lentivirus expressing KLF6, and then the levels of KLF6 mRNA and protein in the cells were measured using qRT-PCR and western blot, respectively. ^#^*P* < 0.05 compared with mimic NC. ^&^*P* < 0.05 contrasted with inhibitor NC. **f**-**h** The percentages of M1 macrophage and M2 macrophage were determined by using flow cytometry. **i** and **j** qRT-PCR was carried out to detect the expression of iNOS and Arg-1 mRNAs. **k** and **l** ELISA assay was used to measure the concentration of IL-1β and CCL17 in the cell supernatant. ^#^*P* < 0.05 compared with mimic NC. ^&^*P* < 0.05 *vs*. inhibitor NC or LV-NC

We predicated the binding sites of human miR-200b and KLF6 3’-UTR using starBase website (Fig. 4a), and proved the combination of miR-200b and KLF6 3’-UTR (Fig. 4b). Moreover, our results showed that increasing of miR-200b significantly declined the levels of KLF6 mRNA and protein, while decreasing of miR-200b markedly upregulated the levels of KLF6 mRNA and protein in M0 macrophages (Fig. 4c-e). Then, we explore whether miR-200b affects macrophage polarization through inhibiting KLF6. M0 macrophages were infected with the lentivirus expressing KLF6 following miR-200b mimic transfection. Our data revealed that the inhibition of miR-200b to macrophage M1 polarization, and the promotion of miR-200b to macrophage M2 polarization were rescued by KLF6 overexpression (Fig. 4f-h). The inhibition of miR-200b to M1 macrophages-related protein iNOS expression, and the promotion of miR-200b to M2 macrophages-related protein Arg-1 expression were reversed by KLF6 overexpression (Fig. 4i, j). Furthermore, our resulted indicated that miR-200b reduced the production of M1 macrophages-related cytokine IL-1β, and facilitated the production of M2 macrophages-related cytokine CCL17 in M0 macrophages, while which were recused following overexpression of KLF6 (Fig. 4k and l). In conclusion, miR-200b promoted macrophage M2 polarization and inhibited M1 polarization through suppressing KLF6.

## Discussion

Exosomes are extracellular lipid bilayer vesicles with a diameter of 30–100 nm, released by almost all cells in physiological and pathological conditions. The exosome isolated from biological fluids including plasma may act as a liquid biopsy to assist the diagnosis of cancer [18]. LAMP-1, TSG101, CD86 and heat shock proteins 70 are the important characteristic molecules. Currently, TEM, NTA and western blot are the major methods for exosome identification [19, 20]. Here, we determined using above methods that we obtained the plasma-derived exosomes successfully. It was known that exosomes play their biological function via carrying numerous molecules, such as protein, miRNA and circRNA, thus to involve in the development of many disorders [21]. For instance, ovarian cancer cells changed the morphology of human peritoneal mesothelial cells (HPMCs) to a mesenchymal phenotype via transferring exosomes to HPMCs. Subsequently, the HPMCs facilitated the invasion of ovarian cancer cells via submitting CD44-overexpressed exosomes to the cancer cells [22]. Furthermore, exosomal miR-99a-5p was highly expressed in the serum of EOC patients. EOC cells-derived exosomal miR-99a-5p could effectively facilitate the invasion of cancer cells through regulating HPMCs by promoting fibronectin and fibronectin, suggesting exosomal miRNA might be a potential target for ovarian cancer diagnose and treatment [23]. In our present study, we found that miR-200b was increased in the plasma-derived exosomes of EOC patients, and explored the action mechanism of miR-200b regulating ovarian cancer cell proliferation and invasion.

Several previous studies have indicated that miR-200b is high expressed in the serum of EOC patients compared with healthy women, and the serum miR-200b level is associated with the grade of EOC [24, 25]. However, a recent research proved that miR-200b is lower expressed in ovarian cancer cells when compared to the human ovarian fibroblasts, and it suppresses the proliferation of cancer cells through targeting PI3K/AKT signaling pathway [26]. The role and action mechanism of miR-200b in ovarian cancer still not clear. M1 macrophages can be induced by interferon-γ, and highly secrete IL-1β, IL-12 and other pro-inflammatory cytokines. M2 macrophages can be induced by IL-4, IL-13 and IL-10, and highly express CCL17, IL-10 and other anti-inflammatory cytokines [27]. The polarization of macrophages toward M1 or M2 is closely related to the progression of cancer. For instance, miR-130a was increased in M1 macrophages, and it was proved to impede the progression of non-small cell lung cancer through promoting the polarization of macrophages toward M1 and inhibiting M2 polarization [28]. M2 macrophages contribute the proliferation of ovarian cancer cells [29]. Besides, it was revealed that inhibiting the differentiation of macrophages into M2 macrophages could effectively suppress the progression of ovarian cancer [30]. In this present study, our data showed that miR-200b inhibited macrophage M1 polarization and facilitated M2 macrophage, thus to promote the ovarian cancer cell proliferation and invasion.

KLF6 is a member of KLFs family, which are the highly conserved zinc-finger proteins. Growing evidence proved that KLF6 acts as a tumor suppressor molecular in multiple malignant cancers [31]. KLF6 was proved to impede macrophages M2 polarization and M2 macrophages-mediated tumor metastasis [32]. In ovarian cancer, some molecules could accelerate the development of the disease through binding with the 3’-UTR region of *KLF6* and then suppressing the expression of KLF6, for instance miR-630 [33]. Here, we found that miR-200b promoted macrophages M2 polarization through suppressing KLF6 expression.

## Conclusions

Overall, our data indicated that miR-200b was increased in the plasma-derived exosomes of EOC patients, and it boosted ovarian cancer cell proliferation and invasion via inducing macrophages M2 polarization by suppressing KLF6 expression. Our results might provide a novel target for ovarian cancer treatment.

## Data Availability

The datasets used and/or analysed during the current study are available from the corresponding author on reasonable request.
